# Seasonal Change in Trophic Niche of Adfluvial Arctic Grayling (*Thymallus arcticus*) and Coexisting Fishes in a High-Elevation Lake System

**DOI:** 10.1371/journal.pone.0156187

**Published:** 2016-05-20

**Authors:** Kyle A. Cutting, Wyatt F. Cross, Michelle L. Anderson, Elizabeth G. Reese

**Affiliations:** 1 Red Rock Lakes National Wildlife Refuge, U.S. Fish and Wildlife Service, Lima, Montana, United States of America; 2 Department of Ecology, Montana State University, Bozeman, Montana, United States of America; 3 Department of Biology, The University of Montana Western, Dillon, Montana, United States of America; National Cheng Kung University, TAIWAN

## Abstract

Introduction of non-native species is a leading threat to global aquatic biodiversity. Competition between native and non-native species is often influenced by changes in suitable habitat or food availability. We investigated diet breadth and degree of trophic niche overlap for a fish assemblage of native and non-native species inhabiting a shallow, high elevation lake system. This assemblage includes one of the last remaining post-glacial endemic populations of adfluvial Arctic grayling (*Thymallus arcticus*) in the contiguous United States. We examined gut contents and stable isotope values of fish taxa in fall and spring to assess both short- (days) and long-term (few months) changes in trophic niches. We incorporate these short-term (gut contents) data into a secondary isotope analysis using a Bayesian statistical framework to estimate long-term trophic niche. Our data suggest that in this system, Arctic grayling share both a short- and long-term common food base with non-native trout of cutthroat x rainbow hybrid species (*Oncorhynchus clarkia bouvieri x Oncorhynchus mykiss)* and brook trout (*Salvelinus fontinalis)*. In addition, trophic niche overlap among Arctic grayling, hybrid trout, and brook trout appeared to be stronger during spring than fall. In contrast, the native species of Arctic grayling, burbot (*Lota lota*), and suckers (*Catostomus* spp.) largely consumed different prey items. Our results suggest strong seasonal differences in trophic niche overlap among Arctic grayling and non-native trout, with a potential for greatest competition for food during spring. We suggest that conservation of endemic Arctic grayling in high-elevation lakes will require recognition of the potential for coexisting non-native taxa to impede well-intentioned recovery efforts.

## Introduction

Introductions of non-native species represent a leading threat to global biodiversity [1,2], with native freshwater fish assemblages left particularly vulnerable [3]. Control of non-native fish populations is oftentimes a primary mission of conservation organizations [4]. However, effective management of non-native fish is difficult due to behavioral and trophic plasticity in open water systems [5,6,7]. Indeed, successful non-native invaders typically occupy a greater diversity of habitats, are active during longer time periods, and show more generalist foraging strategies than native species [8,9], allowing them to thrive across a broad range of environmental gradients, and thus inhabit a large niche space. Further complicating the management of non-native fishes, species-poor native communities may be more vulnerable to invasion than species-rich ones [10–12]. Such factors can lead to an increased competitive advantage for introduced fishes, often to the detriment of native species. Moreover, these interactive competitive pressures may be modulated by seasonal or long-term changes in habitat conditions.

High-elevation, shallow lake ecosystems common at middle to high latitudes are often categorized as “cold polymictic” [13], characterized by extended periods of ice cover and strong seasonal variation in water temperature and oxygen concentrations [14]. These factors potentially influence coexistence of native and non-native species by forcing high spatial or temporal overlap in habitat and food use [15–17]). In these systems, open-water oxygen concentrations vary in response to strong seasonality in ice cover, primary production and ecosystem respiration [18]. Lake oxygen minima usually occurs during winter and spring, a time when surface ice cover is at its annual maximum. Increased ice cover reduces atmospheric oxygen exchange along with physical mixing of the water column. These processes coupled with high rates of organic matter respiration, lowers dissolved oxygen concentrations. During this time, fish stress and mortality may be reduced through physiological adaptations (e.g. metabolic suppression, glycogen stores; [19]) or by aggregating in well-oxygenated waters, including distinct strata or depths in the water column, or stream deltas [20–22]. In summer and fall, daily to weekly water column stratification of temperature and dissolved oxygen is broken down by wind mixing, and fishes tend to be widely distributed throughout the lake. Such large temporal shifts in ice-cover and oxygen could influence intraspecific and interspecific interactions such as competition and predation between native and non-native fishes [17]. Given the likelihood of similar spatial and temporal distributions among fish species in winter and spring, there exists a strong probability of seasonal differences in interspecific competition [17].

Interspecific competition between native and non-native fish species in high-elevation, shallow lakes has been observed through both competition between omnivores for similar food items [23] and predation by one fish species on another fish species [24]. Feeding habits can influence important life history characteristics of fishes; including growth, egg quality and quantity, and timing of spawning that determine the ultimate success of populations [25–27]. A greater understanding of seasonal foraging dynamics could greatly enhance managers’ ability to conserve native fish populations in the face of non-native fish introductions.

Grayling (*Thymallus* spp.) are a circumpolar genus of fish found in coldwater lakes and streams throughout Europe, Asia, and North America. In the contiguous United States, native Arctic grayling (*Thymallus arcticus*) have declined dramatically in distribution and abundance since European settlement, and are now limited to several rivers and lakes in the upper Missouri River drainage of Montana [28,29]. This decline is likely due to several factors including cattle grazing impacts to riparian habitat, irrigation withdrawals, dams and other barriers (e.g. culverts), and interactions with introduced non-native species [30–33]. While Arctic grayling of the upper Missouri River drainage are not currently listed under the Endangered Species Act, conservation concerns remain for this rare and endemic species [29].

The coexistence of Arctic grayling and several non-native fish species in shallow, high elevation lakes of the upper Missouri provide an opportunity to assess potential interspecific competition for this rare and endemic fish species. We investigated seasonal diet breadth and degree of trophic niche overlap in the fish community of a single large lake during fall and spring seasons. These contrasting seasons, with strong variation in water temperature and oxygen concentrations, offer the opportunity to identify if potential competition for food use is greater during the spring, than is during the fall season. We used gut content analysis to quantify short-term (i.e. days) diets and degree of trophic niche overlap among fish taxa. We then used these short-term dietary estimates as priors in a Bayesian stable isotope mixing model to further assess trophic niche overlap and the potential for competition [34]. Stable-isotope (e.g. δ^15^N and δ^13^C) analysis of muscle tissue can effectively assess diet, trophic position, and trophic niche overlap over a relatively long-time (i.e. few months) span, commensurate with the period of muscle growth and turnover. Thus, muscle tissue can effectively assess the ‘diet history’ of fishes prior to a sampling event [35]. Finally, we contrasted feeding habits, as estimated using stable isotopes, collected during spring and fall, to evaluate how dietary breadth and degree of trophic niche overlap differs across seasons. Our research represents a critical first step in identifying potential mechanisms governing coexistence of Arctic grayling and other fish species in this seasonally variable system, while providing important natural history information for fishery managers and decision makers.

## Materials and Methods

### Ethics Statement

We conducted this study under fish collection permits (27–2011 and 18–2011) graciously issued by Montana Fish, Wildlife, and Parks, which covered fish collections and release and sacrifice, including method of sacrifice.

#### Study Area

Native adfluvial-dwelling Arctic grayling are currently limited to very few locations in the contiguous United States. Several populations persist in the Centennial Valley that is located in the Greater Yellowstone Ecosystem, including lakes and streams within Red Rock Lakes National Wildlife Refuge. Historic records dating back to the early 20^th^ century indicate that large numbers of Arctic grayling were present in Centennial Valley lakes, and successful spawning occurred in twelve tributary streams across the Centennial Valley [36]. However, recent sampling efforts suggest that only two of these streams are currently used for spawning by Arctic grayling, with both located within Red Rock Lakes National Wildlife Refuge [36]. One stream (Odell Creek) is thought to contain few (*n* < 50) actively spawning adults, while another stream (Red Rock Creek) has seen large fluctuations in numbers of documented spawners over several decades [30,33]. To date, information on the trophic ecology is currently lacking for this lake-dwelling population of Arctic grayling and other fishes during the fall and spring.

Our study was conducted in Upper Red Rock Lake, a high-elevation waterbody located in the Red Rock Lakes National Wildlife Refuge of southwestern Montana (44°40ˊ N, 111 °47ˊ W, 2030 m elevation). Upper Red Rock Lake occupies a shallow, postglacial depression that forms the largest wetland complex in the Greater Yellowstone Ecosystem [36]. The lake encompasses approximately 893 ha and has a maximum depth of 2 m. It is classified as cold polymictic due to an average summer water temperatures of >4°C, a lack of consistent thermal stratification in the summer and continual ice cover for up to 7 months a year from November to April [36]. Due to a prolonged winter and spring season, low oxygen conditions can persist for months. Non-lethal threshold effects from hypoxia exist for freshwater salmonids, and are much higher than the reported lethal threshold (<2 mg/L). Davis (1975, [37]) identified an incipient oxygen response threshold, the point at which physiological and behavioral effects first become apparent, as being 6.0 mg/L for freshwater salmonids. Schreck et al. (1997, [38]) describes fish behavioral changes in stressful habitats may significantly affect an individual’s ability to survive through reductions in food acquisition, predator avoidance, and habitat selection. In our lake system, we measured oxygen concentrations from 8 January to 2 April 2011 using a YSI Model 55 Dissolved Oxygen Meter and found concentrations averaged 3.9 mg/L (± 0.4 SE) over this period. We assume these low oxygen concentrations are common across most years in our high-elevation lake system due to consistently severe winter and spring conditions.

### Field Sampling of Fish and Prey Items

We collected Arctic grayling and other dominant native fish (i.e., burbot [*Lota lota*], suckers (white [*Catostomus commersonii*] and long-nose [*Catostomus catostomus*])), as well as non-native salmonids (brook trout [*Salvelinus fontinalis*], and cutthroat-rainbow trout hybrids [*Oncorhynchus clarkia bouvieri x Oncorhynchus mykiss*] hereafter “hybrid trout”) for analysis of dietary composition and stable isotopes on multiple occasions in fall (2–19 October) and spring (1–26 May) in 2011 and 2012. During each season, we collected fish from 3–4 locations that represented both mid-lake and stream outlet habitats. To collect fish across all adult and subadult age and size classes, we used a combination of two standard gill nets (100 m length; mesh size: 7.6 cm) and one experimental gill net (38.5 m length) containing 5 panels of graduated mesh sizes (i.e. between 1.9 and 5.1 cm). On each sampling occasion, we set nets perpendicular to shore at dusk and retrieved after a relatively short period (4 hours) to avoid Arctic grayling mortality. Combining all data from all nets set in each season, we calculated catch-per-unit-effort (CPUE: fish/net hour) for each species of fish during both spring and fall seasons to provide a proxy for relative abundance.

We also collected dominant macroinvertebrates from multiple locations in Upper Red Rock Lake in May and October of 2012, a period that overlapped with 2012 fish collections. We collected benthic macroinvertebrates with dip nets (500 μm mesh) and a boat-deployed bottom sled (1 mm mesh; Wildco Wildlife Supply Company, Yulee, Florida, USA). We sorted samples in the field by eye into taxonomic groups, placed into separate cryogenic vials, and froze for subsequent stable isotope analysis. During sample sorting, we placed voucher specimens in 95% ethanol for more detailed taxonomic identification. We collected pelagic invertebrates (largely cladocerans) at the benthic sample locations with a plankton net (153 μm mesh; Wildco Wildlife Supply Company) towed at mean depth for ~2–3 minutes. We collected samples of filamentous algae (and associated diatoms) and detritus from the same locations as invertebrates.

### Laboratory Sample Preparation

We collected stomach contents of Arctic grayling using non-lethal gastric lavage [39] and monitored fish stress prior to release to maximize post-handling survival. We sacrificed other fish taxa via a quick blow to the head disabling brain function, following which the alimentary canal was removed. We immediately preserved all stomach contents in ethanol. We also removed a muscle biopsy sample (3.5 mm diameter) from each individual for subsequent isotope analysis. We froze isotope samples at -20°C prior to analysis. Across the two year study, we collected 176 muscle tissue samples during spring (Arctic grayling: *n* = 34; hybrid trout: *n* = 38; brook trout: *n* = 6; sucker: *n* = 60; burbot >450mm: *n* = 14; burbot <450mm: *n* = 24) and 155 muscle tissue samples during fall (Arctic grayling: *n* = 21; hybrid trout: *n* = 52; brook trout: *n* = 5; sucker: *n* = 67; burbot >450mm: *n* = 9; burbot <450mm: *n* = 1). Since we captured only one small burbot (<450 mm) during fall, we did not include small burbot in our isotope analyses during this season. Fish muscle biochemistry reflects the long-term average diet (few months, [40,41]) of fishes, therefore isotope values of fish muscle collected during spring represented a period between late-winter and spring seasons. Similarly, fish muscle tissue collected during fall represented the average, long-term, diet during late-summer and fall. To simplify this, we refer to these different times as “spring” and “fall” in subsequent interpretation of our isotope results, unless otherwise stated.

We prepared fish stomach contents for identification in the laboratory by rinsing onto a 63-μm metal sieve to remove contents out of the alimentary canal. We identified all small dietary items, generally to genus, placed in separate aluminum dishes, dried at 55°C, and weighed to determine dietary contributions by weight. Some of the diet material, while clearly from a plant or animal, was unidentifiable and placed in a general ‘other’ category. In addition, many fish contained amorphous detrital material that we could not clearly identify. We assumed this amorphous material represented a mixture of ‘basal resources’ (i.e., largely detritus, but containing some algae, [42]). Before analysis, we combined many fine-scale diet categories to simplify our interpretation (e.g., multiple families within Ephemeroptera were combined). Given that fish prey was typically partially digested, we did not identify these samples to species, and instead lumped all fish prey into a single ‘fish’ category. However, based on specimens we observed to be routinely least digested, it appears that the dominant species of fish prey consumed by predators were small burbot (<200mm in length).

To prepare tissue samples for isotope analysis, we rinsed each sample with deionized water (to remove any surface debris), freeze-dried, and chopped into small pieces using fine-tipped scissors. Because carbon isotope (δ^13^C) values may be influenced by variation in lipid concentration [43], we divided each fish sample into two parts, extracting lipids from one part using a 24-h 2:1 chloroform:methanol solution [44] and retaining the second part with lipids for comparison and nitrogen (δ^15^N) isotope analysis. We similarly divided most invertebrate samples in half, and extracted lipids from one part using the same extraction technique. For a given taxon, we used the difference between the extracted and non-extracted samples as a correction factor for those samples that were not lipid-extracted. We weighted both lipid and non-lipid extracted samples to ~1mg in tin capsules, crushed, and analyzed independently for δ^13^C and δ^15^N using continuous flow isotope-ratio mass spectrometry (Thermo Scientific Delta V, Center for Stable Isotopes, University of New Mexico, Albuquerque, New Mexico, USA). Stable isotope values are reported in parts per thousand (‰) relative to standards for δ^13^C (Vienna PeeDee Belemnite) and δ^15^N (atmospheric [AIR] nitrogen). Our estimated analytical error for δ^13^C and δ^15^N was ±0.04‰ and ±0.1‰, respectively, based on replicate within-run measurements of laboratory organic standards.

### Data analysis

In an attempt to account for ontogenetic shifts in diet, we used logistic regression to quantify the size at which piscivorous fish switched their diet from <50% to >50% fish prey by weight as determined by gut content analysis. This analysis revealed that only one taxon, burbot, exhibited such an ontogenetic diet shift, and this shift occurred at a length of ~450 mm. We therefore treated small (<450 mm) and large (>450 mm) burbot as separate groups in all analyses. This analysis likely did not account for subtle ontogenetic dietary shifts for some size classes of certain taxa of fish. Instead, our focus was on general broad interspecific patterns of piscivory rather than fine-scale intraspecific patterns. We used a one-way ANOVA with a Tukey Honestly Significant Difference test to assess seasonal variation in isotope values of muscle tissues of fish in 2011 and 2012. Prior to analysis, we tested the data for homogeneity of variance and normality of the residuals and confirmed to meet the assumptions of ANOVA.

To quantify dietary overlap between fish taxa, we used Schoener’s Index (*D*, [45]):

$$D=1-\;0.5\;\overset{n}{\underset{i=1}{\sum }}\left(\left|p_{ij}\;-\;p_{ik}\right|\right)$$

where *p*_*ij*_ and *p*_*ik*_ are the relative proportions, based on dry weight, of prey item _*i*_ in the diets of species _*j*_ versus species _*k*_. In order to calculate Schoener’s Index, we first determined the relative proportion of a given prey item (_*i*_*)* within the gut contents of an individual, given a particular fish species (_*j*_*)*. We then averaged these proportions across all individuals for a given fish species. This index varies between zero (no overlap) and one (complete overlap) and enables a quantitative estimate of trophic niche overlap between taxa, or size classes. Typically, values greater than 0.6 are interpreted as a significant degree of dietary overlap between taxa.

We used a bootstrapping technique [46] to generate medians and 95% confidence intervals for dietary overlap from each pair of species or size classes. During each bootstrap iteration, we sampled individuals of each taxon with replacement to calculate mean dietary proportions. These means are used to generate estimates of Schoener’s Index for each species pair. We repeated this process 1,000 times to produce 1,000 values of Schoener’s index, as well as bootstrapped medians and 95% confidence intervals derived from these vectors.

To estimate dietary contributions to fish, we used a Bayesian stable isotope mixing model [47]. This model accounts for variation in isotope values of different food sources, percent carbon and nitrogen concentrations of food sources, and diet-to-muscle tissue isotopic discrimination. We omitted prey items from the isotope mixing model that were either not found in gut contents or were minor (<6%) contributors to fish diets. In addition, we included results on short-term trophic niche (from gut content analysis) as prior information into the Bayesian isotope mixing model to further refine and constrain our estimates of long-term dietary contributions [47]. We estimated isotopic dietary endmembers within the mixing model from 73 samples representing 10 aquatic taxa during spring, and 51 samples from 7 aquatic taxa during fall. Our fish prey endmember was based on isotope values of small fish collected during netting efforts (<283mm; i.e. Arctic grayling, burbot, and suckers) in 2011 and 2012. Because we collected basal resources (algae and detritus) during only the fall of 2012, we have considerable uncertainty about this endmember. Thus, we used the average values of both algae and detritus to represent our ‘basal resource’ endmember within the isotope mixing model. We assumed isotopic discrimination between diet to fish consumer to be 0.4 ± 1.3‰ SD for δ^13^C and 3.4 ± 1.0‰ for δ^15^N [48].

For each fish species, we quantified isotopic niche breadth (i.e., size of isotope ellipse) and proportional isotopic niche overlap with other taxa using standard ellipse areas corrected for small sample size (SEA_c_; Siber routine [48], Siar package [47]). Ellipse breadth measured for individual populations help elucidate the foraging tendencies of different taxa within a community and allow a quantitative comparison of core niche similarity among fish taxa [49]. We also compared overlap of ellipse areas between fish taxa (as a percentage of the smaller ellipse between two species) during each season. We evaluated differences in interspecific niche size using the posterior distribution of the standard ellipse areas (SEA_B_, [49]). For our ellipse area analysis, we were more interested in seasonal differences than interannual differences, thus we combined fish isotope data within the same season from both 2011 and 2012. This was justified by the relatively small interannual difference in isotope values of fishes within a given taxon, and our focus on inter-seasonal trophic niche.

In addition to our ellipse area analyses, we measured trophic diversity [50, 51] to gain additional inference regarding fish trophic ecology. Trophic diversity within a species is the average of the Euclidean distances of individual δ^13^C and δ^15^N values from the isotopic centroid for a given fish population. Trophic diversity is influenced by either consuming a variety of prey items that differ isotopically, or by the variability in isotope values of prey items. Because metrics presented in Layman *et al*. (2007, [50]) are sensitive to sample size, we constructed and compared trophic diversity across species and seasons by bootstrap resampling individuals with replacement (10,000 iterations) to produce a vector of mean metric values and 95% confidence intervals. For each bootstrap iteration, we based sample size on the fish species with the lowest number of samples in either fall or spring. All statistical analyses were conducted using R 3.1.1 [52].

## Results

### Fish Community Assemblage

**Fig 1:**
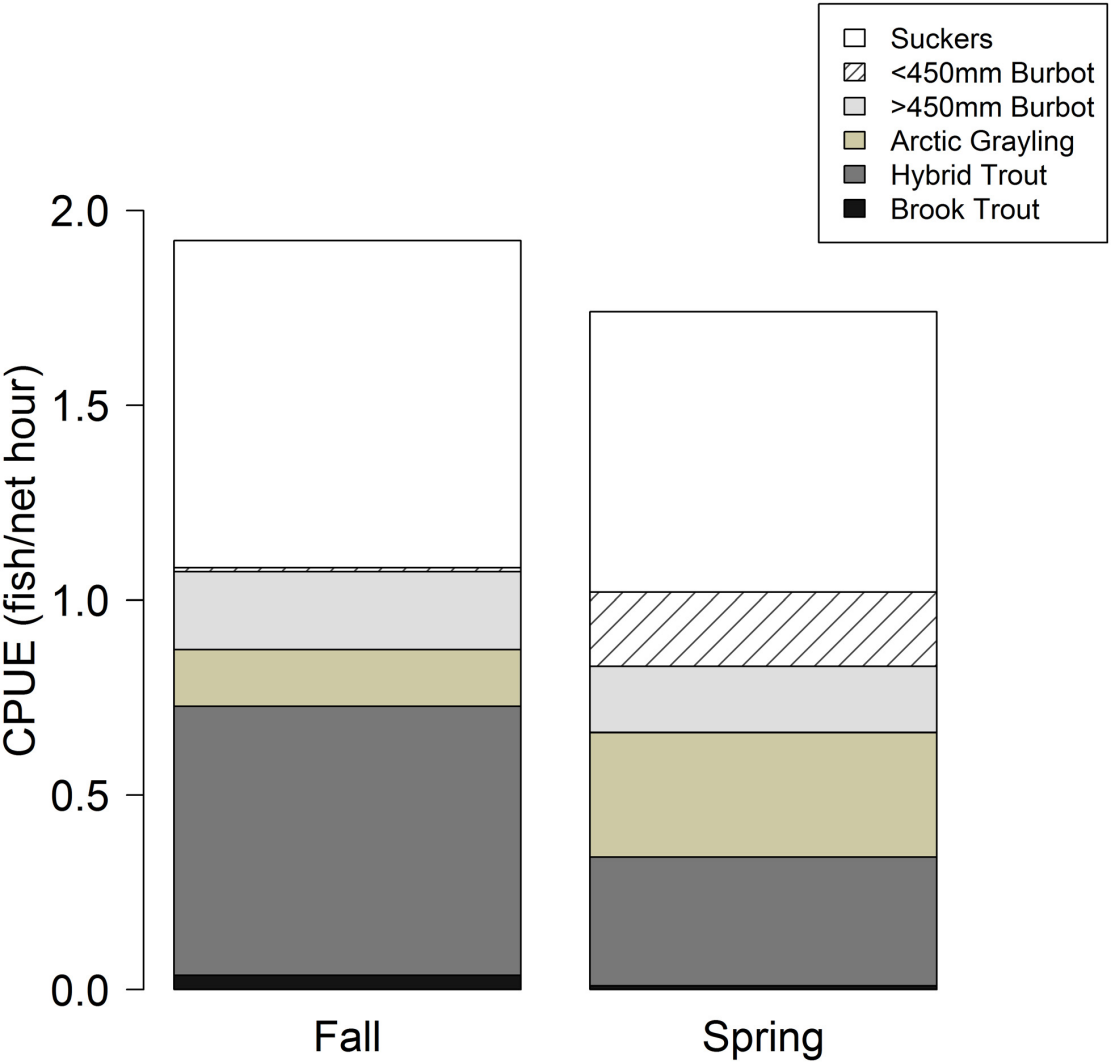
Standardized mean catch-per-unit-effort (CPUE) from the fish community during spring and fall, 2011–2012, Upper Lake, Red Rock Lakes National Wildlife Refuge, Montana, USA.

Average CPUE for the entire fish community was similar during fall (1.9 fish/net hour) and spring (1.7 fish/net hour). Suckers comprised the majority of total fish captured during spring (41%) and fall (44%), followed by hybrid cutthroat trout (spring: 19%, fall: 36%). Arctic grayling comprised a higher proportion of the total catch during spring (19%) than fall (8%). Whereas the proportion of large (10%) and small (11%) burbot captured was similar during spring, while only large burbot were captured during fall (10%). Brook trout represented the smallest proportion of total CPUE during both seasons (Fig 1).

### Fish Diet Composition

**Fig 2:**
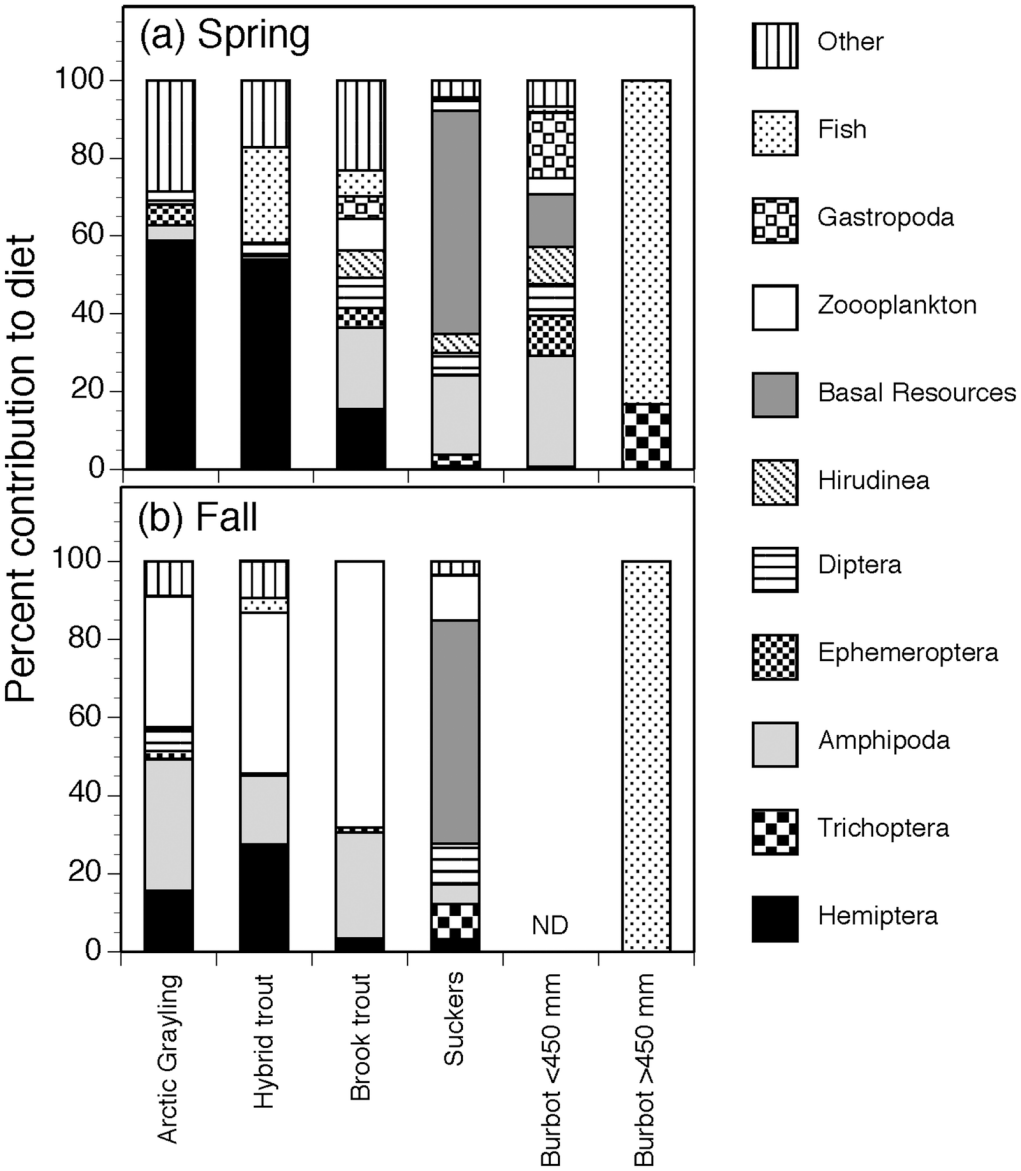
Diet composition by percent weight of the fish community in spring (a) and fall (b). Burbot were separated into two ontogentic classes based on the contribution of fish to their diet.

Proportion of prey in gut contents varied among taxa and across seasons, but diets of Arctic grayling were generally more similar to non-native salmonids (brook trout and hybrid trout) than either burbot or suckers (Fig 2, Table 1). In the spring, Hemiptera, specifically Corixidae, dominated the diets of Arctic grayling (58%) and hybrid trout (54%), resulting in a high level of dietary overlap (*D* = 0.72) between these taxa (Fig 2, Table 1). Although diets of brook trout were more diverse than Arctic grayling, Hemiptera still contributed 14% to their overall diet, resulting in high dietary overlap (*D* = 0.77) with grayling (Fig 2, Table 1). Diets of suckers were diverse and consisted of basal resources (57%), amphipods (20%), Diptera (6%) and leeches (5%; Fig 2). Suckers exhibited modest dietary overlap with Arctic grayling, small burbot, and brook trout (Fig 2, Table 1). Diet composition of small burbot (<450mm) showed relatively low dietary overlap with Arctic grayling, hybrid trout, and brook trout (*D* < 0.23; Fig 2, Table 1), but was somewhat similar to the diet of suckers (*D* = 0.47, Table 1), including basal resources (14%), amphipods (30%) and gastropods (17%). Diet of large burbot (>450mm) differed considerably from other fish taxa (*D* < 0.22) throughout the spring, which consisted exclusively of fish (83%) and Trichoptera (17%).

**Table 1:** Schoener’s index (1970,[45]) of trophic niche overlap among fishes. Values represent bootstrapped medians and 95% confidence intervals. ‘−’ signifies no data.

***Spring***	Arctic grayling	Hybrid trout	Brook trout	Suckers	Burbot <450 mm	Burbot >450 mm
Arctic grayling	X	0.72 (0.58–0.84)	0.77 (0.53–0.92)	0.35 (0.23–0.47)	0.29 (0.19–0.38)	0.00 (0.00–0.01)
Hybrid trout		X	0.64 (0.43–0.81)	0.22 (0.14–0.31)	0.23 (0.14–0.32)	0.22 (0.09–0.37)
Brook trout			X	0.40 (0.20–0.65)	0.23 (0.14–0.33)	0.00 (0.00–0.00)
Suckers				X	0.47 (0.35–0.57)	0.04 (0.00–0.07)
Burbot <450mm					X	0.02 (0.00–0.53)
Burbot >450mm						X
***Fall***	Arctic grayling	Hybrid trout	Brook trout	Suckers	Burbot <450 mm	Burbot >450 mm
Arctic grayling	X	0.70 (0.55–0.85)	0.64 (0.51–0.76)	0.33 (0.20–0.49)	−	0.00 (0.00–0.00)
Hybrid trout		X	0.62 (0.46–0.77)	0.29 (0.16–0.46)	−	0.04 (0.00–0.11)
Brook trout			X	0.19 (0.09–0.31)	−	0.00 (0.00–0.00)
Suckers				X	−	0.00 (0.00–0.00)
Burbot <450mm					X	−
Burbot >450mm						X

Fall diets of Arctic grayling, brook trout, and hybrid trout were measurably different than those during spring, while the diets of burbot and suckers remained similar between seasons (Fig 2, Table 1). Zooplankton became more prominent constituting 34%, 41%, and 68% of diet in Arctic grayling, hybrid trout, and brook trout, respectively. These three fish species consequently exhibited a large degree of dietary overlap (*D* > 0.62). Large burbot consumed exclusively fish during fall (100%), whereas suckers consumed a diversity of prey, including basal resources (57%), Diptera (10%), zooplankton (12%), Trichoptera (9%), and amphipods (5%). Similar to the spring, large burbot and suckers showed low dietary overlap with the other fish taxa.

### Food web structure

**Fig 3:**
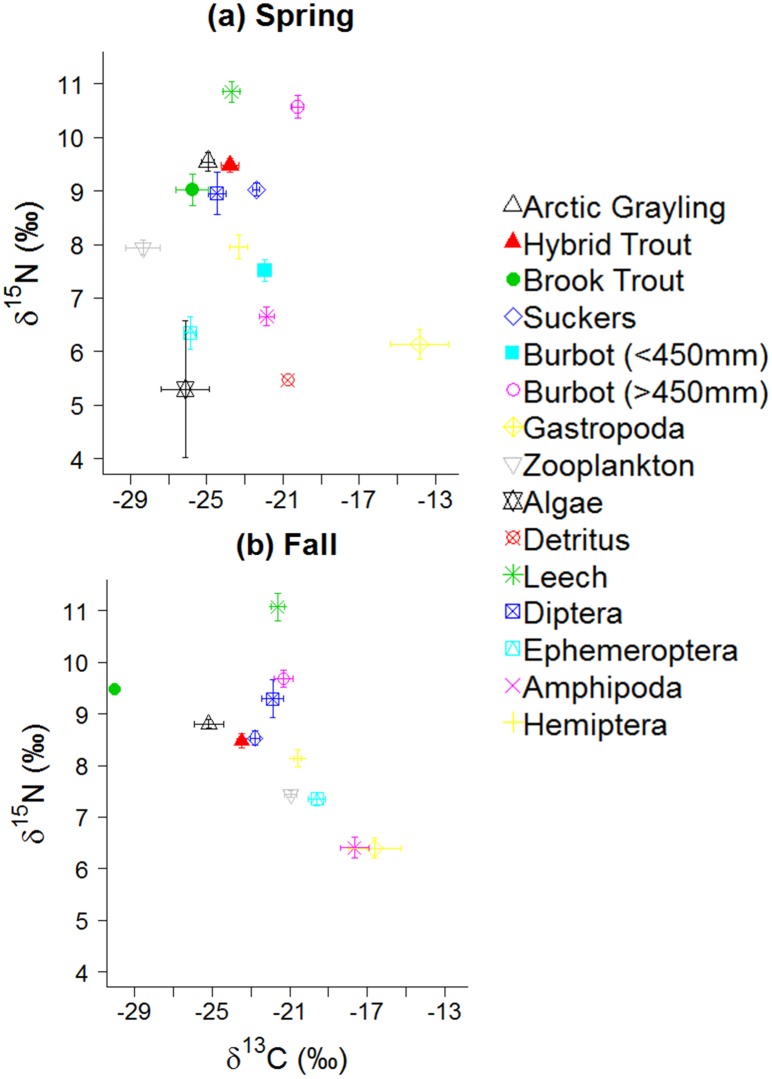
Stable carbon and nitrogen isotope values of food web components in spring (a) and fall (b) of 2012. Symbols represent means ± 1SE.

Primary producers (algae) and detritus, representing the basal trophic position, exhibited the lowest δ^15^N values (Fig 3). During both seasons, invertebrates and fishes occupied mid- and high-range δ^15^N values, respectively, with the exception of leeches, which showed consistently higher δ^15^N values than fishes. In general, amphipods, Ephemeroptera, and gastropods showed less-enriched δ^15^N values than zooplankton, chironomids, and Hemiptera. δ^13^C values of invertebrates and fishes were largely bracketed by algae and detritus during spring, except for zooplankton and gastropods, which fell outside the range of sampled basal resources. The range in δ^13^C values of prey sources was lower during fall than it was in spring (Fig 3).

**Fig 4:**
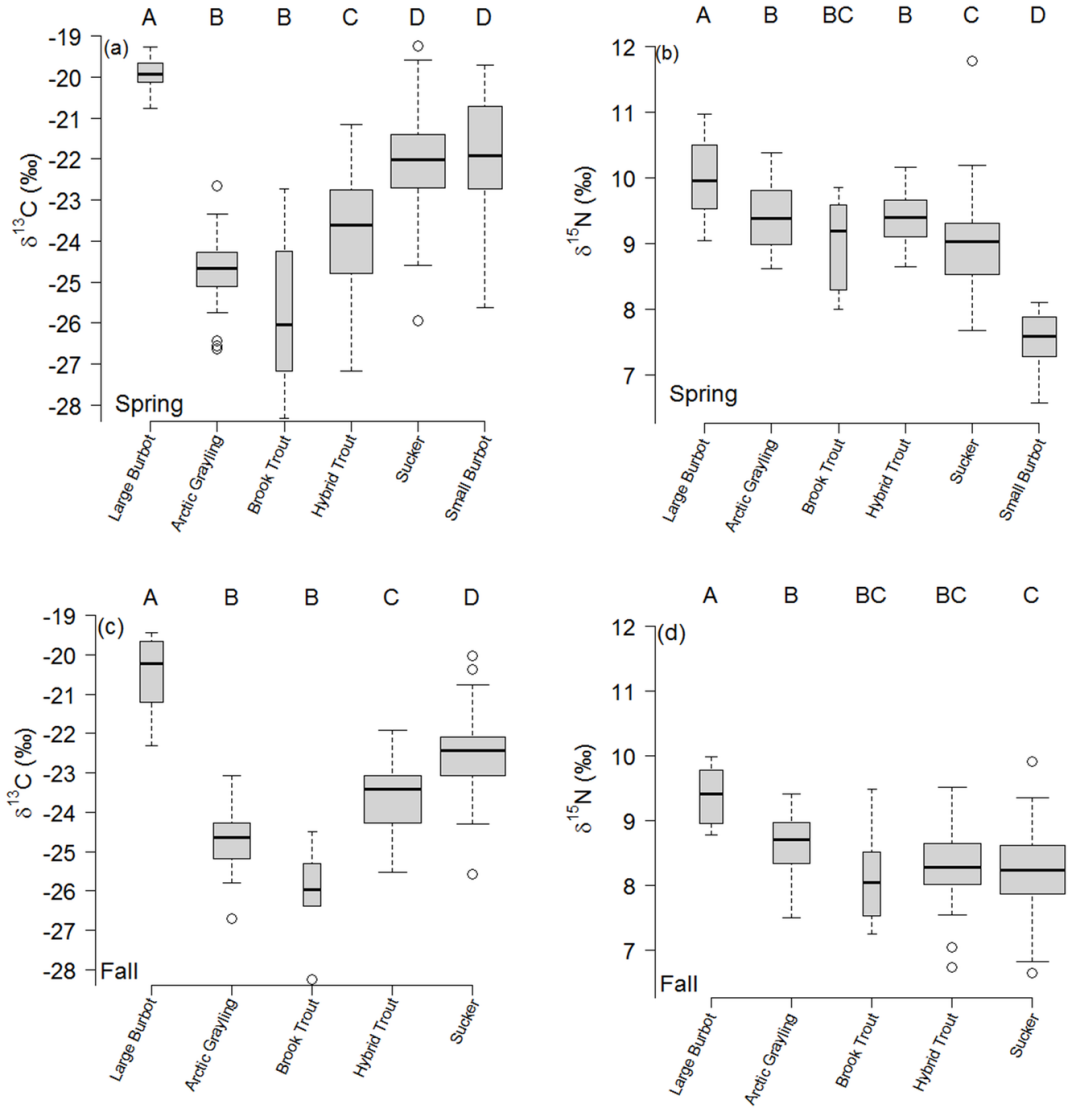
Boxplots indicating isotopic comparison for spring (a and b) and fall (c and d) of 2011 and 2012. The horizontal line depicts average isotope values, boxes indicate standard errors, and the whiskers indicate standard deviation. Dots represent outliers. Unique letters represent significant differences determined by Tukey Honestly Significant Differences.

Analysis of variance demonstrated significant differences in average δ^13^C and δ^15^N values during spring and fall for most fish species (spring - δ^13^C: *F*_5,170_ = 52.51; δ^15^N *= F*_5,170_ = 50.17; fall - δ^13^C: *F*_4,149_ = 43.16; δ^15^N: *F*_4,149_ = 9.82; *P* < 0.001; Fig 4); however, isotope values of Arctic grayling consistently overlapped that of hybrid and brook trout. Spring and fall δ^13^C values were similar between Arctic grayling and brook trout indicating similarity in consumed prey items. Consistent δ^15^N values show that seasonal trophic position remained similar among Arctic grayling, brook trout, and hybrid trout (Tukey Honestly Significant Difference all *P* > 0.05).

**Fig 5:**
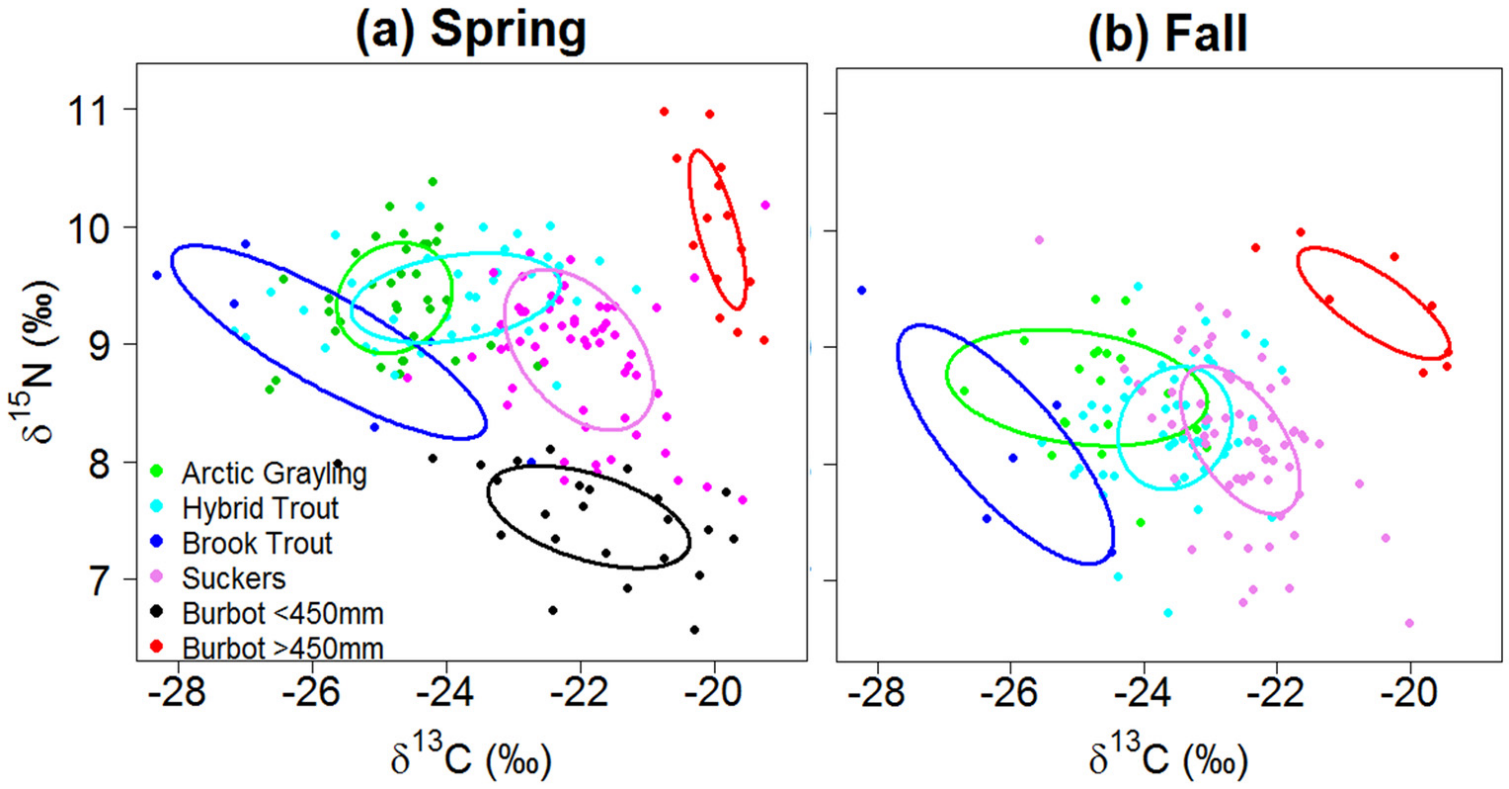
Stable isotope Bayesian ellipses (solid lines) depict trophic niche breadth and overlap in the spring (a) and fall (b) based on SEA_c_ analysis for Arctic grayling, large burbot (>450 mm), small burbot (<450 mm), hybrid trout, sucker, and brook trout. Each point represents an individual fish. Only one small burbot was captured in the fall, preventing quantification of population niche metrics.

Trophic niche overlap among fish species can be related to the magnitude of potential competition [53,54]. During spring, trophic-niche breadth, as estimated by standard ellipse areas, was largest for brook trout (3.20 ‰^2^), intermediate for Arctic grayling, hybrid trout, and burbot <450mm (1.22–2.10 ‰^2^), and smallest for large burbot (Fig 5, Table 2). During fall, niche breadth was largest for brook trout and Arctic grayling, intermediate for sucker, hybrid trout, and smallest for small burbot. (Fig 5, Table 2). Trophic niche overlap was greatest between Arctic grayling and non-native salmonids, and this overlap was greatest during spring (23–64%). Arctic grayling, however, exhibited no trophic niche overlap with suckers, or small and large burbot during spring, (Fig 5, Table 2) while hybrid trout showed slight overlap with suckers during the same period (15%; Fig 5, Table 2). During fall, patterns of dietary overlap were broadly similar to spring, but Arctic grayling showed slight overlap with suckers (9.3%), while trophic niche overlap was twice (33%) as great between hybrid trout and suckers.

**Table 2:** Trophic diversity for fish taxa (median values along with 95% confidence interval’s derived from bootstrapping), small sample size-corrected standard ellipse areas (SEA_c_), and comparison in SEA_c_ overlap between pairs of species (as % of isotope niche space) for spring (top matrix) and fall (bottom matrix). ‘− ‘ signifies no data. Trophic diversity values derived from Layman (2007, [50]).

***Spring***	**Trophic Diversity**	***SEA***_***C***_ ***(‰***^***2***^***)***	***Arctic grayling***	***Hybrid trout***	***Brook trout***	***Sucker***	***Burbot <450mm***	***Burbot >450mm***
Arctic grayling	0.78 (0.56–1.02)	1.22	X	63.5	22.9	0.0	0	0
Hybrid trout	1.28 (0.92–1.66)	1.78		X	5.7	15.2	0	0
Brook trout	1.57 (0.56–2.42)	3.20			X	0.0	0	0
Sucker	0.99 (0.71–1.39)	2.10				X	0	0
Burbot <450mm	1.12 (0.52–1.92)	1.78					X	0
Burbot >450mm	0.61 (0.39–0.84)	0.58						X
***Fall***	**Trophic Diversity**	***SEA***_***C***_ ***(‰***^***2***^***)***	***Arctic grayling***	***Hybrid trout***	***Brook trout***	***Sucker***	***Burbot <450mm***	***Burbot >450mm***
Arctic grayling	1.19 (0.62–2.17)	2.96	X	45.4	14.6	9.3	0	–
Hybrid trout	0.83 (0.61–1.04)	1.39		X	0	32.9	0	–
Brook trout	1.49 (0.39–2.60)	4.43			X	0	0	–
Sucker	0.87 (0.63–1.16)	1.47				X	0	–
Burbot <450mm	–	–					X	–
Burbot >450mm	0.93 (0.59–1.23)	1.08						–

**Fig 6:**
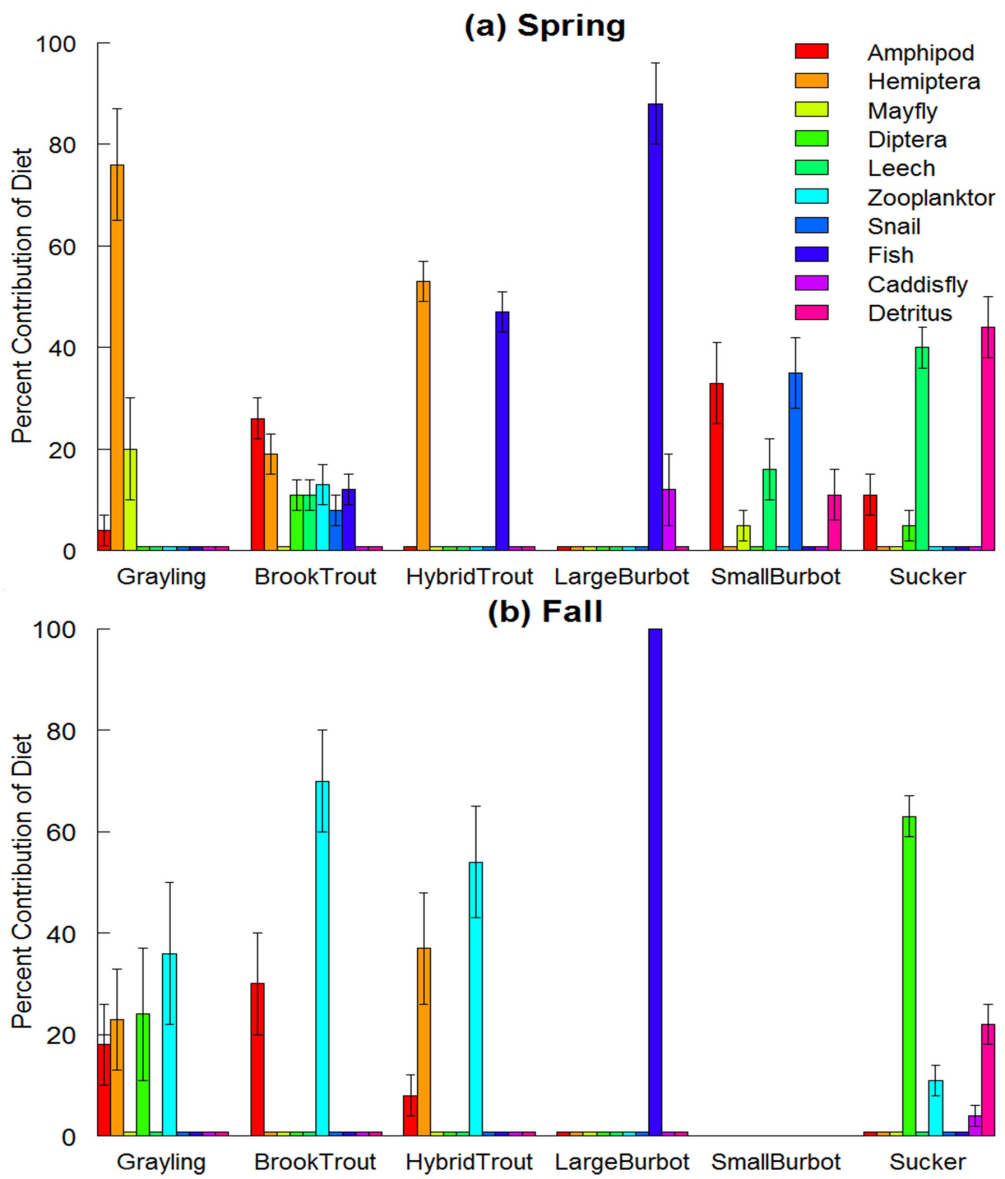
Contributions (% mean ± 1 SE) of dietary sources to fishes during spring (a) and fall (b) based on a two-isotope (δ^15^N and δ^13^C) Bayesian mixing model with informative priors (Parnell *et al*., 2010; [47]).

Results from the isotope mixing model suggest that Arctic grayling and non-native salmonids share similar prey items over long-time spans. Arctic grayling primarily consumed Hemiptera and Ephemeroptera during spring, while they consumed a greater diversity of prey items during fall (i.e., zooplankton, Diptera, Hemiptera, and amphipods; Fig 6). In contrast, brook trout utilized a relatively wide array of prey items including Hemiptera during spring, but focused predominantly on zooplankton and amphipods during fall. Hybrid trout were largely supported by Hemiptera during both seasons, but switched diets from fish during spring to zooplankton during fall (Fig 6). Small burbot also consumed a diversity of prey during spring, while large burbot consumed fish and Trichoptera during the same season (Fig 6). In contrast to the other fish species, suckers showed a strong dependence on basal resources during both seasons, while leeches dominated their diet during spring and Diptera during fall.

Trophic diversity was estimated imprecisely for most fish species during both spring and fall (Table 2). Confidence intervals for trophic diversity overlapped each other for all fish species during both seasons, with the exception of hybrid trout and large burbot during spring. During spring, individual hybrid trout exhibited a greater trophic diversity than large burbot (Table 2).

## Discussion

Intentional or inadvertent introduction of non-native fishes to lakes and streams across the world has been a critical agent of ecological change, with vast consequences for species diversity, food web interactions, and ecosystem processes [55–60]. In a large number of cases, introduced fish taxa have been extremely successful and now dominate fish assemblages in terms of abundance, biomass, and productivity [61,62]. In this study, we utilized a multisource Bayesian stable isotope mixing model informed by gut content analysis of fish [34] to quantify trophic niche and potential for competition in a shallow, high-elevation lake fish assemblage (Fig 6). Our analysis shows that the native Arctic grayling population shares a high degree of dietary overlap with non-native hybrid trout and brook trout (Figs 2 and 5). In this system, hybrid trout represent the second most common species (Fig 1), suggesting strong potential effects on the persistence of the less common Arctic grayling since the introduction of hybrid trout to the Upper Lake watershed in 1970s [63].

Niche differentiation of competitors is believed to result, in part, from coevolutionary processes [64]. Minimal overlap in diet occurred among native fish species at our study site, possibly representing evolution of divergent life history strategies that mediate competition [65, 66]. The low diversity of native fishes observed in our high-elevation system is typical of middle to high latitude lakes across the world, as influenced by lake surface area, depth, climate variability, and glacial history [67]. Diverse life history adaptations, such as iteroparity, diet flexibility, habitat generalization, cannibalism, and migration, are commonly observed in high latitude lakes as mechanisms for reducing competition in climatically extreme lake environments [68, 69]. In contrast, our results suggest a strong potential for competition between non-native salmonids and endemic Arctic grayling, especially during spring when dietary overlap were strongest (Figs 2, 5 and 6, Table 1). We believe that greater dietary overlap during this period may be related to low temperatures and oxygen availability, coupled with reduced production of algae and invertebrates during this time of year. These environmental conditions are expected to reduce availability of suitable habitat, thus increasing local densities of fish in areas with favorable temperature, oxygen and food [22, 70, 71] The influence of competition for food, coupled with a stressful physical environment during winter and spring months, can result in depletion of fish energetic condition in trout [72]. The effects of seasonal resource depletion on fish condition may be more severe in lakes with higher fish density, and such effects may be especially detrimental to cold water-adapted fish due to increased competition for limited resources during winter and spring [70]. Climate change is also projected to change resource availability through increases in water temperature [73], leading to increases in eutrophication and reduced oxygen concentration of small-lake systems [74]. Shifts in fish body size, fish community composition, and declines in Arctic charr (*Salvelinus alpinus*) populations across shallow lakes in Europe have already been linked to recent climate change and increasing water temperatures [75]. Given the fact that Arctic grayling and hybrid trout in this cold water system are spring spawners [33], our results along with projections of future climate change will have important consequences for allocating energy reserves in preparation for migration and spawning [16]. Increased dietary overlap with non-native salmonids during winter and spring could reduce survival or overall body condition in Arctic grayling, and could act as a cross-seasonal effect on reproductive fitness during the subsequent spring spawning season [25].

The inter-seasonal changes in dietary breadth have been documented in other lake systems in response to dietary shifts [17, 68, 76–78] and habitat fragmentation [50]. We detected increases in dietary breadth from spring to fall in Arctic grayling, brook trout, and large burbot. In contrast, dietary breadth declined for hybrid trout and suckers across the same seasons. For Arctic grayling, brook trout, and hybrid trout, the change in dietary breadth was driven largely by an increase in Hemiptera during the spring, while zooplankton and amphipods dominated their diets during fall (Fig 6). Hemiptera (specifically Corixidae) may be more available than other aquatic insects in the frozen lake during winter and spring, as they store oxygen bubbles suspended in the water column under the wings and around the abdomen [79]. The ability to store oxygen makes Corixids well suited for survival during periods of low oxygen [79]. Reduced dietary breadth for Arctic grayling during winter and spring could be driven by unsuitable habitat conditions; forcing grayling to use small patches of suitable habitat where a reduced prey community remains [71]. Nykänen *et al*. (2004, [71]) found that Arctic grayling in northern Finland remained in the same microhabitat patches throughout the winter and spring months, potentially exposing them to a limited set of prey items. Whereas during fall, Arctic grayling, hybrid trout, and brook trout increased their consumption of amphipods. Consumption of amphipods, a high-energy food source, could help with reproduction in brook trout (a fall spawner), and for survival of all species by acquiring and storing somatic lipid reserves as preparation for the prolonged winter season.

While we have shown strong evidence for trophic niche overlap between Arctic grayling and co-existing salmonids, it is important to recognize that our data do not provide direct evidence for interspecific competition, *per se*. In order to demonstrate competition, there must be evidence of resource limitation (*sensu* [80,81]), and we did not assess the availability of food relative to the demand by different fish taxa. In practice, there are a few ways to assess whether Arctic grayling populations are indeed competing with non-native taxa. The first requires quantifying the productivity of prey items, and comparing prey production, over a given time period, to the amount of prey required by fishes over that same period. While the energetic demands of fish may be assessed through bioenergetic models [82], prey production is typically estimated via labor- and time-intensive sampling (e.g., [42,65,83]) that is often beyond the scope of most projects and budgets (but see [84]). An alternative is to conduct field removals of hypothesized competitors while following the population-level response of the focal taxon. In the case of Upper Red Rock Lake, a five-year non-native removal program was initiated in 2013 to test hypotheses about competition and predation between Arctic grayling and hybrid trout. Even though the current fish removal does not target native burbot, a piscivorous species in later life-history stages, their population could increase due to release of prey with the removal of thousands of non-native hybrid trout. This potential population response by burbot could not only lead to increased rates of cannibalism on their own early life-history stages, but also of predation on Arctic grayling and other native fish species. Continued monitoring of Arctic grayling throughout this experiment should help provide complementary support for the potential of competition and predation in this freshwater lake system.

Arctic grayling were once common and abundant across the northern contiguous United States, but are now limited to only a few locations where populations remain at risk due to land use, non-native taxa, and climate-related changes to flow and temperature regimes [28,29]. Lake-dwelling Arctic grayling in Montana are the last stronghold for endemic Arctic grayling across the contiguous United States, making them a critical component of the nation’s biodiversity [29]. Our study elucidates the importance of seasonal short- and long-term trophic niche, and diet overlap and breadth to native and non-native fish taxa in one of their last strong hold of Upper Red Rock Lake. Understanding the trophic interactions and complex foraging strategies of Arctic grayling at the southern end of their range in the United States has implications for grayling management elsewhere (Canada, Russia, Europe), and can help conservationists develop science-based plans on how to manage non-native species. We also show that dietary overlap between Arctic grayling and densely populated non-native salmonids was greatest during the spring season. Future work is now needed to assess interspecific differences in vulnerability to predation or other mortality factors that mediate interspecific competition during spring in both Arctic grayling and other fish species inhabiting high-elevation lakes.
